# Do all trainers think the same? Exploring use of the Think Aloud method to understand colonoscopy trainer thought processes

**DOI:** 10.1055/a-2633-9032

**Published:** 2025-07-23

**Authors:** Amy Whitehead, Joe Causer, Melissa Rankin, Kristin McGinty-Minister, Paul O'Toole

**Affiliations:** 1 4589 Sport and Exercise Science, Liverpool John Moores University, Liverpool, United Kingdom of Great Britain and Northern Ireland; 2 4589 School of Nursing and Advanced Practice, Liverpool John Moores University, Liverpool, United Kingdom of Great Britain and Northern Ireland

**Keywords:** Quality and logistical aspects, Training, Quality management

## Abstract

**Background and study aims:**

Colonoscopy is a complex skill to teach. Understanding trainer cognition is an important factor in determining the level of trainer competence. This study aimed to explore colonoscopy trainer thoughts as they observed trainee performance using the Think Aloud method (TA).

**Methods:**

Eleven trainers verbalized their thoughts (TA) while watching three video recordings of trainees performing a colonoscopy procedure. TA audio was transcribed verbatim and content was analyzed to determine valence of verbalizations, frequency of verbalized themes, and level of verbalizations in relation to depth of response.

**Results:**

Descriptive differences were observed between trainers, highlighting a lack of consistency in relation to their thoughts while observing a trainee perform colonoscopy.

**Conclusions:**

This study provides support for use of TA as a method to understand trainer cognition in colonoscopy. It suggests the need for further research to explore consistency of training across trainers.

## Introduction

Colonoscopy is a complex task, requiring interplay of advanced motor and cognitive skills. This skillset results from rigorous training and experience to meet competency requirements for certification that are set by the Joint Advisory Group on Gastrointestinal Endoscopy (JAG). Over the past two decades, the mode of training for endoscopy in the UK has evolved from a traditional informal process in the endoscopy unit that parallels apprenticeship 1 to a blended competency-based approach, incorporating an evidence-based training framework 2 and relevant technological advances such as computer simulator training and e-learning tools. This approach has led to a formal educational pathway for endoscopy trainers (train-the-trainers) 1 . It is acknowledged, however, that there is still a need for further evolution of training pathways, and that additional training and support for trainers is required 2 3 4 .

Evidence suggests that endoscopy trainers are akin to sports coaches, facing similar issues when looking to improve performance 5 . Developing expertise in many sports requires acquisition, retention, and application of highly complex practical skills performed under pressure. Clear parallels exist with the endoscopy learning experience. Essential factors for practical skills training include practice, demonstration, motivation, instruction, and feedback 5 .

Conscious competence is seen as a key factor for the trainer in colonoscopy 1 . Like skilled athletes, experienced endoscopists can perform colonoscopy quickly and efficiently without conscious thought about each step in the procedure, but they may struggle to explain how and why particular maneuvers are successful. An explicit understanding of technical aspects of the procedure, and the ability to deconstruct actions, is critical if trainers are to provide useful instruction to trainees. Waschke et al *.* 1 highlight how widespread lack of conscious competence is a fundamental barrier to effective colonoscopy training. A colonoscopy trainer’s understanding of the procedure should allow them to recognize and anticipate the technical challenges their trainee is facing and be able to offer useful suggestions to overcome or prevent them. This is largely based on careful observation and interpretation of the visual information available. Despite the central importance of this process, we have little understanding of what trainers attend to during colonoscopy, the factors determining why and when they intervene, and how they decide what to discuss in feedback. Furthermore, methods to explore this have not been considered.

A potential approach for understanding these underpinning thought processes is the Think Aloud (TA) method. TA originated in the field of cognitive psychology and aims to understand how people think by requiring participants to verbalize their thoughts while performing a task 6 . The method is becoming popular within coaching and sports psychology 7 8 9 ; has previously been employed in nursing 10 and other medical settings 11 ; and has been used to develop a competency framework for colonoscopy 12 .

The TA method could be a useful tool to investigate about how trainers think, process information, and make decisions about a training episode. It might also be useful for trainer development. This study aimed to explore use of TA with colonoscopy trainers during observation of video-recorded colonoscopy procedures.

## Methods

### Participants

Eleven colonoscopy trainers were recruited to take part. All had completed the JAG Train the Colonoscopy Trainers (TCT) course between 2000 and 2021 and had participated as faculty on Basic Skills in Colonoscopy (BSC) training courses (with a range of 8 to 50 courses). Three trainers were clinical (nurse) endoscopists, the others all consultant gastroenterologists (2 female, 9 male). Participants were labelled P1 to P11 and ranked according to training experience: P1–4 were TCT faculty, P5–8 were experienced BSC course faculty, and P9–11 were less experienced (had taught on fewer than 10 BSC courses).

### Ethics statement

Ethical approval was granted from the authors’ research institute ethics committee (ethics number: 20/SPS/040).

### Procedure

Prior to data collection, all participants received a TA training video including instructions on how to conduct TA 13 . Data were collected via Microsoft Teams. Participants watched three video recordings (without audio) that showed the insertion phase (from rectum to caecum) of colonoscopy procedures performed by three different endoscopists. They were told these procedures were being performed by trainee colonoscopists.

Participants were instructed to TA and given the following instructions: “Please think aloud anything that comes to mind as you are watching the trainee. Please also verbalize any specific feedback you may give the trainee while watching the video”.

The videos included a picture-in-picture view of the magnetic endoscopic imager output (Scopeguide - which provides a virtual representation of the scope position) and a view of the endoscopist’s hands. Neither the patient nor the endoscopist could be identified.

Recordings were selected to represent different training challenges.

Video 1 (8 minutes long) was a straightforward procedure performed by an experienced consultant gastroenterologist. This was chosen to represent the challenge of finding useful performance enhancing feedback for an advanced trainee.

Video 2 (16 minutes) was performed by an endoscopy fellow who had trained outside the UK without the benefit of Scopeguide. He struggled to resolve persistent sigmoid looping and took a long time to negotiate the splenic flexure. It represented the challenge of dealing with a trainee who lacks insight into colonoscopy dynamics.

Video 3 (14 minutes) was a very difficult procedure performed by an experienced colonoscopist. There was complex, atypical sigmoid looping; the challenge was to interpret the Scopeguide image correctly and offer suggestions for loop resolution.

### Data analysis

Each participant’s TA audio was transcribed verbatim and transcripts ranged from 1434 words to 7785 words, with an average of 5490 words.

A post-positive epistemology informed data analysis. A team approach was used, whereby three authors engaged in content analysis. The JAG Colonoscopy Direct Observation of Procedural Skills (DOPS) assessment tool is widely used for evaluating trainee competence 14 . It contains eight domains focusing on technical aspects of the procedure, each of which is accompanied by detailed grade descriptors. These were used to generate a system for classifying the verbalizations according to themes ( Table 1 ). The DOPS domains “Visualization” was combined with “Air Management” for convenience, and a “General Observations” category was introduced for comments which could not be classified according to the other themes.

**Table:** **Table 1** Coding framework for trainer Think Aloud data.

Themes	Description	Details	Example quote
**General**	General observations about	Relating to the procedure, anatomy, or pathology.	“The mucosa is inflamed here”
	skill level or overall approach	Relating to trainee’s general approach.	“He’s quite good, just needs better refinement really”
	Predictions	Predictions about what might happen next.	“Just about to pop round the splenic flexure”
**Scope handling**	Left hand technique	Relating to operation of control body.	“Left hand position is nice”
	Body/arm positioning	Relating to operator’s position or left/right arm actions.	“He's kind of leaning forward a little bit. He doesn't need to do that”
	Ergonomics	Comments about stance/positioning/scope handling in relation to risk of repetitive strain injury.	“He holds the umbilicus inside the arm, which is going to make it difficult to get to the left and right wheels”
	Shaft management	Management of loops outside the patient.	“Lots of external looping going on, which doesn't help”
	Use of variable stiffener	Comments about appropriateness of variable stiffener application (except when directly related to looping).	“OK, there's lots of use of the stiffener again”
	Use of right hand on controls	Single handed *vs* 2-handed technique.	“Using two hand steering. I would try to discourage that.”
**Tip control**	Luminal awareness	How quickly operator identifies lumen if it is lost.	“So, the lumen is up at 1 o'clock and this trainee’s doing quite well”
	Tip steering	Speed and accuracy of tip deflections.	“He's actually got reasonable tip control. Believe it or not!”
	Torque steering	Maintenance of appropriate torque on shaft.	“Not sure why we've turning into the wall there”
	Integrated technique	Combines controls and torque steering smoothly.	“He’s thrashing around all over the place”
**Insufflation**	Degree of insufflation	Comments about over or under insufflation.	“Look how much air he’s used. So we’ve got a big, inflated bowel.”
**and**	Gas vs water	Choice of water or gas to open up lumen	“I would have probably used water a little bit more”
**Visualization**	Management of fluid/feces	Actions taken in response to fluid/feces	“Nice. They’re getting the fluid to the 6 o'clock position for suction.”
	Washing lens	Maintenance of a clear view using lens washing	“Clean your lens there”
	Adequacy of view for advancement	Comments relating to the luminal view and actions taken to optimize views	“We haven't got a clear luminal view. I'd be telling them to pull back so they could see where the lumen is”
**Strategy/problem solving**	General approach	Shows awareness of strategies useful in specific situations and applies them quickly	“That straightening has gone so well; it's actually moved the tip forward. So, he's done that very well”
	Anticipation of difficulty	Shows awareness of challenges and responds proactively to prevent or minimize difficulty	“He’s applied anti-clockwise here to keep the scope against the back of the abdomen”
	Use of position change	Uses position change pro-actively to prevent difficulties	“They’ve just done a pre-emptive position change”
		Prompt decision to change position when appropriate Selects position changes in a logical sequence	“They’ve moved the patient early, which is great” “He's rolled the patient to left lateral, which is completely illogical”
	Use of external pressure	Uses external pressure to overcome or prevent difficulty when appropriate	“Good abdominal pressure there and it’s working for him, which is great and that’s worked very well”
	Strategy modification	Changes strategy if clearly not working	“Definitely need to do something or we'll just hover in the same area”
**Loop management**	Loop prevention	Takes measures to prevent looping	“He’s pulled back so that the scope is straight so well done”
	Recognition of looping	Responds appropriately to presence of loop	“He's gone onto a really long scope position and just pushed through”
	Resolving loops	Resolves loops promptly and completely	“He’s finally got that sigmoid loop out, so he succeeded in the end”
	Pushing through loops	Appropriateness of decision to push through loops	“It was a poor decision at the start to try and resolve the loop early”
**Pace and progress**	Speed of insertion	Advances scope at appropriate speed for conditions	“I would probably be asking him to slow right down now here”
	Speed of movements	Movements too quick or too slow	“If he just took his time, he might be a bit more efficient”
	Progress	Comments about rate of progress	“Making nice steady progress”
**Safety and comfort**	Patient comfort	Comments about likely impact of actions on patient comfort	“Patient must be getting quite uncomfortable by now”
	Observations of patient	Comments about whether patient is showing signs of comfort/pain	“Looks like the patient’s getting uncomfortable, by the way she's holding her tummy”
	Excess looping/local pressure.	Concerns about degree of looping or local pressure	“So the loops formed, the patient might be feeling a bit of discomfort”
	Response to hazard/difficulty	Response of operator to perceived hazards	“He's in the diverticular segment, so needs to be certain where the lumen is”
	Blind advancement	Pushes blindly around bends	“Pushing blindly here. Doesn't appear to have any awareness of where the lumen is”
	Response to pathology	Response of operator to pathology that increases risk	“We’ve got severe colitis here, so I’d pause the procedure at this point”

Verbalizations were classified by valence: that is, whether the individual thoughts were positive, negative, or neutral ( Table 2 ). Where a single verbalization contained a balance of both positive and negative themes, it was classified as neutral.

**Table:** **Table 2** Coding framework for valence of Think Aloud data.

Valence	Description
Negative	Observations that operator’s technique or approach is not ideal Observations about sub-optimal room set up or ergonomics Suggestions that trainee should have done something differently, or sooner Observations that a maneuver or technique has not worked Concerns expressed about possible hazards or discomfort Negative comments about luminal views, lack of progress or scope position Negative thoughts about how the trainer is feeling themselves Negative thoughts about the video quality or study design
Neutral	Neutral observations or thoughts related to the procedure or process. Statements about where the trainer’s attention is focused Verbalization of questions the trainer would be asking themselves Expressions of lack of certainty - not related to operator’s performance Thoughts that contain a balance of positive and negative elements
Positive	Positive thoughts about operator’s technique, approach, or competence Positive comments about room set-up or ergonomics Positive comments about progress, views, or scope position Suggestions that the trainer would offer praise or commendation Recognition that a maneuver or technique has worked Recognition that comfort and safety are being managed appropriately Positive thoughts about how the trainer is feeling Non-specific expressions of approval

It was observed that varying degrees of insight were displayed by verbalizations. Some verbalizations were simple observations about what was being witnessed; others involved analysis of why certain problems were arising and suggestions for resolving them; and, finally, some comments related to overall strategic approach to colonoscopy, or discussed how the observer might interact with the trainee in a genuine training situation. As a result, levels of verbalization were generated ( Table 3 ).

**Table:** **Table 3** Levels of verbalization.

Level 1	Observation	Requires understanding of good basic colonoscopy technique
Level 2	Analysis	Requires understanding of strategies necessary to overcome difficulties
Level 3	Synthesis	Requires high-level understanding of the colonoscopy training process

Individual participant data were then descriptively compared and presented for level of verbalization, valence and content of TA.

## Results

### Level of verbalization (%) per participant

**Fig. 1:**
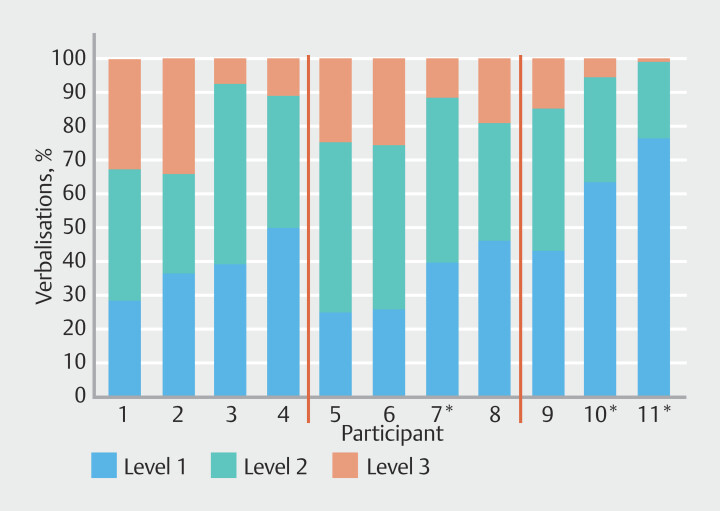
Percentage of each participant’s verbalizations classified by level. Red lines separate P1-P4 (TCT faculty), P5-P8 (BSC Faculty), P9-P11 (less experienced). *Nurse practitioner.

**Fig. 2:**
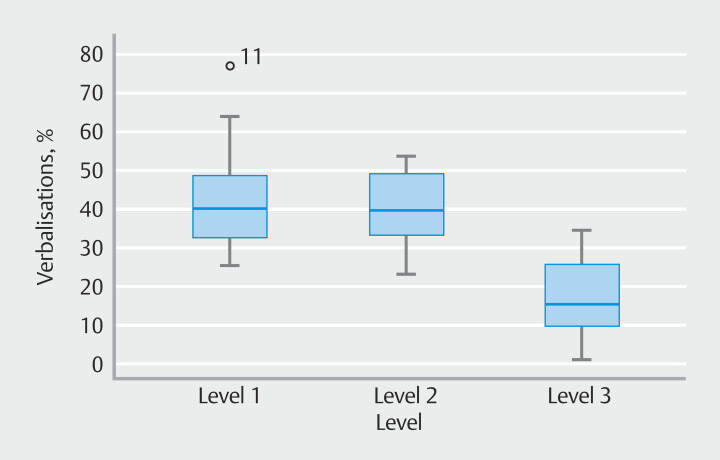
Boxplot of the percentage verbalizations classified by level. o = mild outlier.

Fig. 1 shows the large variability in levels of verbalizations used by each participant across the three videos. There was a wide distribution of verbalizations for level 1 (25–77%), level 2 (23–53%), and level 3 (0.8–34%). Fig. 2 shows that median level 1 verbalization was 40% (interquartile range [IQR] 28%-50%). Participant 11 was a mild outlier at 77%. Median level 2 verbalization was 39% (IQR 31%-49%). Median level 3 verbalization was 15% (IQR 8%–26%). These data show that participants used fewer level 3 verbalizations than level 2, or level 1 verbalizations.

### Valence (%) per participant

**Fig. 3:**
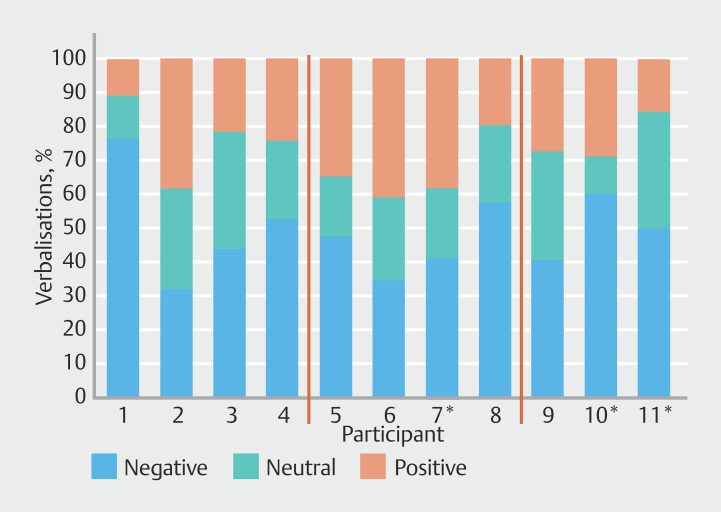
Percentage of each participant’s verbalizations classified by valence. Red lines separate P1-P4 (TCT faculty), P5-P8 (BSC Faculty), P9-P11 (less experienced). *Nurse practitioner.

**Fig. 4:**
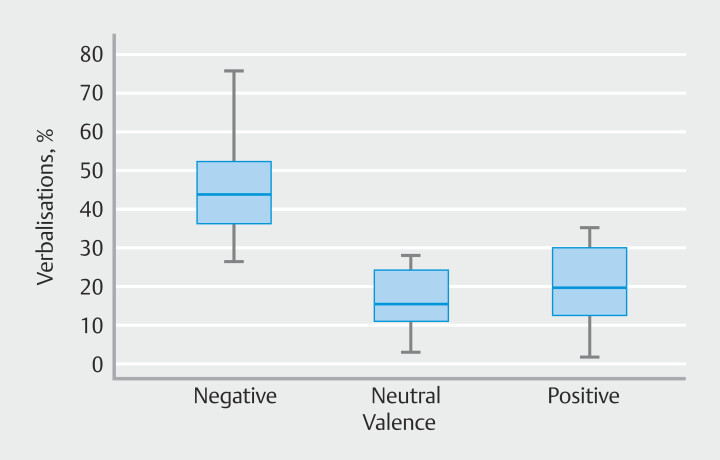
Boxplot of the percentage verbalizations classified by valence.

Fig. 3 shows the large variability in valence used for each participant across the three videos. There was a wide distribution in verbalizations for negative (32%-77%), neutral (12%-34%), and positive (10%-41%). Fig. 4 shows that median negative verbalization was 48% (IQR 41%-58%). Median neutral verbalization was 23% (IQR 18%-32%). Median positive verbalization was 27% (IQR 19%-38%). These data show that participants used more negative verbalizations than positive, or neutral verbalizations.

### Content of TA (%) per participant

**Fig. 5:**
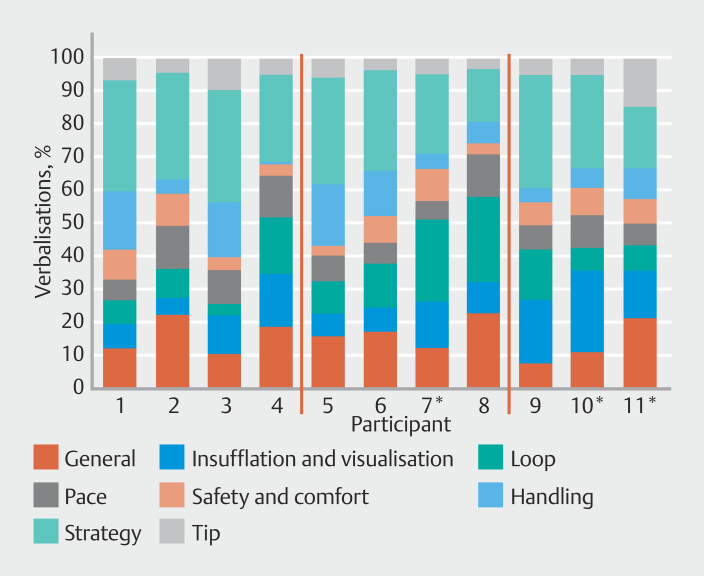
Percentage of verbalizations coded by each think aloud element for each participant. Red lines separate P1-P4 (TCT faculty), P5-P8 (BSC Faculty), P9-P11 (less experienced). *Nurse practitioner.

**Fig. 6:**
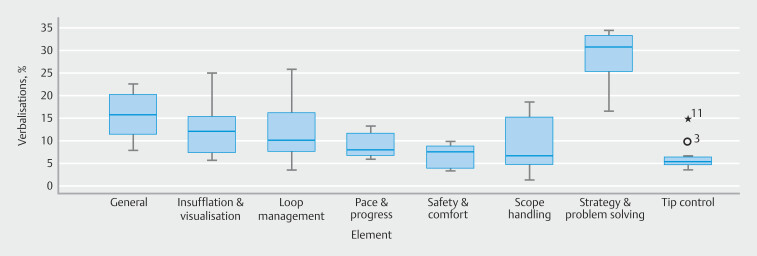
Boxplot of percentage verbalizations coded by each think aloud element. ° = mild outlier, * = extreme outlier.

There was a large amount of variability in TA elements verbalized across the three videos between the participants ( Fig. 5 ). There were high ranges of verbalizations for general (15%), insufflation and visualization (20%), and loop management (21%). Much lower ranges were reported in pace and progress (8%), and safety and comfort (7%). Fig. 6 shows that the highest median was for strategy and problem-solving verbalizations at 31% (IQR 24–34). Scope handling (median = 7%) had the largest IQR (4%-17%). Median tip control verbalization was 5% (IQR 4%-6%). Participant 3 (10%) was a mild outlier, and participant 11 (15%) was an extreme outlier.

## Discussion

This study aimed to explore use of TA with colonoscopy trainers and gain insight into their thought processes during observation of video-recorded colonoscopy procedures. The findings provided evidence that TA is a potentially viable tool for understanding the thought processes of endoscopy trainers.

Every trainer has their own style, so differences in TA between trainers was expected, but perhaps not to the degree demonstrated in this study. The proportion of positive and negative thoughts expressed by each participant varied widely. It is important to remember that trainers were asked to verbalize anything that came to mind, and they were obviously aware the trainee could not hear them. In a real training situation, trainers’ verbalizations would be very different. Nonetheless, their thoughts are likely to influence, to some degree, how individual trainers approach the real training situation. More junior or inexperienced trainers may experience more feelings of being overwhelmed and anxiety 15 . This accounted for some of the negative thoughts verbalized by our least experienced trainers.

The data provide evidence of some differences in focus between participants. For example, TCT courses emphasize the importance of correct scope handling as the basis for good technique. This was a fairly common theme of TA for some trainers but mentioned much less by others, and hardly at all by one participant. Waschke et al. 1 highlighted the importance of teaching consistency across instructors in their white paper guidance on the important principles of training endoscopy teachers.

The tendency to express concerns about patient safety and comfort also showed variation. Focus of attention may be influenced by the trainer’s past experience and training background. For example, Van Oostveen et al. 16 found that surgeons and nurses differ in their perceptions of patient care intensity. They attributed these differences to professional role and daily work activities.

Although the study was not designed to address the impact of training experience on TA content, some speculation is possible. A major focus of BSC courses is to instruct trainees regarding principles of loop management. It was notable that trainers whose training activity centered on the BSC course tended to verbalize thoughts on this theme more often than those with less course experience. The most experienced trainers also made relatively few explicit references to loop management but tended to express similar thoughts in a more sophisticated way, classified under “strategy and problem solving”.

Effective trainers have explicit knowledge and can deconstruct tasks and understand each element of the procedure (“conscious competence”). They are able to analyze the performance of trainees objectively and teach the necessary skills by verbalizing sequential steps effectively to the trainee 1 . Differences in “level” of verbalization among our subjects might reflect differing levels of conscious competence. For example, we would expect experienced trainers to express proportionally more “high-level” thought content (Level 3). Trainers with less experience may be in the cognitive stage of training 17 , where their thoughts are more directed toward simple observations (Level 1). Those with more tacit knowledge will also verbalize thoughts around what they see but may also offer more detailed analysis and evaluation of what they are thinking as they observe the trainee. Although this appeared to be true at both extremes of the experience spectrum, for the majority of trainers, the relative amounts of Level 1, 2, and 3 content showed variation that did not seem to correlate with experience. This is something that needs further exploration.

Not all trainers need to be capable of highly sophisticated analysis of trainee performance. When working with beginners, Level 1 descriptions or Level 2 explanations may suffice, whereas for those assisting more advance trainees, a Level 3 understanding of scope dynamics, ergonomics, and strategy may be more important. Using TA within the Train-the-Trainer course might allow faculty to understand the “level” at which novice trainers are thinking in relation to their trainees, and define the training setting to which they are best suited. This insight might allow Train-the-Trainer faculty to tailor their approach to each delegate.

In a survey of UK trainers 4 , a prominent theme was a desire for feedback from peers and other expert trainers, to provide trainers with insight into their teaching skills. This is best provided by direct observation of training skills in a live situation, but work constraints make such opportunities very difficult to arrange. By reviewing their thoughts while watching a video-recorded training session, the “Think Aloud” method may offer an alternative approach for trainers to reflect on their training skills and style, especially if given the opportunity to compare their thoughts with those of others. Within sport coaching, TA has been used as a reflective practice tool to promote “thinking about thinking” and support coaches in developing their intrapersonal, interpersonal and professional knowledge 9 18 . This work has demonstrated how TA can support coaches to be more aware of and develop their communication skills, understand their athletes better, and increase awareness of their own biases, which in turn allows them to become more effective coaches.

### Limitations

We aimed to explore what trainers were thinking while watching a trainee. Using video recordings created an artificial situation and the verbalizations we recorded do not, of course, reflect what trainers would say if they were actually in the procedure room. Furthermore, TA used in this way examines only cognitive aspects of trainer skill, ignoring many other attributes of good training, such as the ability to develop rapport, communicate clearly, and show support. However, our method would not have worked in a live situation because trainers would have censored their thoughts in the presence of the trainee and patient.

This study was conducted in the UK, where Scopeguide is a standard tool for training 19 . Our participants, therefore, received visual information about looping and scope position within the colon while watching the videos. Trainers who do not regularly use Scopeguide rely on more subtle observational cues to gather this information; these cues may be less reliably conveyed through video-recorded procedures. This could limit the international applicability of our approach.

The analysis conducted within this study relied on the researchers’ interpretations and classification of TA content, based on DOPS domains which overlap to some degree. Researchers may consider multi method approaches and consider both TA data and trainer interpretations of their own data using additional methods such as stimulated recall 20 . Further, researchers may consider adopting alternative epistemological lenses to these data and take a more qualitative approach to data analysis to allow for a more detailed narrative of trainer cognition. This would allow exploration of the more nuanced individual differences observed. Finally, although alluded to in places, we did not investigate expertise differences explicitly within this study. We recommend that future research consider differences in both expertise and professional background (e.g. nurse endoscopists vs medical endoscopist).

## Conclusions

In summary, this study provides a novel insight into the thought processes of colonoscopy trainers using the TA method. Our study highlights differences in the way trainers direct their attention while watching colonoscopy, and the degree to which they analyze and synthesize what they see. This has important implications for colonoscopy training and offers possibilities for use of TA in trainer development.
